# Ratio of matrix metalloproteinase-2 to -9 is a more accurate predictive biomarker in women with suspected pre-eclampsia

**DOI:** 10.1042/BSR20160508

**Published:** 2017-03-15

**Authors:** Hao Feng, Li Wang, Min Zhang, Zhiwei Zhang, Wei Guo, Xietong Wang

**Affiliations:** 1 Department of Obstetrics and Gynecology, Shandong Provincial Qianfoshan Hospital affiliated to Shandong University, Jinan 250014, China; 2 Department of Obstetrics and Gynecology, Provincial Hospital affiliated to Shandong University, Key Laboratory of Birth Regulation and Control Technology of National Health and Family Planning Commission of China, Maternal and Child Health Care of Shandong Province, Jinan 250014, China

**Keywords:** matrix metalloproteinase, preeclampsia, pregnancy, predictive biomarker, receiver operating characte

## Abstract

Pre-eclampsia (PE) is a condition unique to pregnancy, and abnormal expression of matrix metalloproteinases (MMPs) has been implicated in its pathogenesis. We aimed to evaluate the reliability of plasma levels of MMP-2, MMP-9 and their relative ratio in predicting PE. A total of 318 women with suspected PE were recruited for the study, who were subsequently either cleared or diagnosed of PE and grouped accordingly. Their baseline characteristics were compared. Blood samples were also collected from all participants, to determine the plasma levels of MMP-2 and MMP-9. The predictive values of levels of MMP-2 and MMP-9, as well as their ratio, were analyzed using the receiver operating characteristic (ROC) curve. Either MMP-2 or MMP-9 alone did not exhibit any obvious differences between normal and PE pregnancies. However the ratio of MMP-2/MMP-9 was significantly higher in PE-affected pregnancy than normal control group. ROC curve analysis also indicated that the MMP-2/MMP-9 ratio provided better compromise between specificity and sensitivity in distinguishing PE from normal pregnancies, than either of the two MMPs alone. MMP-2/MMP-9 ratio is a more accurate biomarker to predict PE than either MMP-2 or MMP-9 alone.

## Introduction

Pre-eclampsia (PE) is a complicated syndrome that occurs in approximately 2–5% of pregnancies throughout the world [1]. It also contributes to nearly 20% of maternal mortality and morbidities, as well as preterm birth, perinatal deaths, and intrauterine growth restriction [2]. PE is usually characterized by proteinuria and hypertension in the clinic, and its pathogenesis currently still remains elusive [3]. It has been suggested that PE may be caused by poor placental perfusion initiated from shallow cytotrophoblastic invasion and incomplete uterine spiral artery remodeling, which could lead to placental ischemia [4]. Dysregulation of several plasma factors, such as matrix metalloproteinases (MMPs), have been thought to contribute to the insufficient invasion of trophoblast cells and failure of spiral artery remodeling, which eventually result in widespread dysfunction in the maternal endothelium [5].

MMPs are a class of structurally related but functionally diversified zinc- and calcium-dependent endopeptidases [6]. Widely expressed in different tissue types, they exert functions in the remodeling and physiological homeostasis of the extracellular matrix, in order to facilitate various developmental processes. During pregnancy, abnormally expressed MMPs have been reported to cause hypertensive disorders [7]. Specifically in the pathogenesis of PE, various studies have indicated that dysregulated MMPs could adversely alter the vascular remodeling process [3,4,7]. Of particular interest to our present study, two members of the MMP family, namely MMP-2 and MMP-9, are frequently implicated as key factors that contribute to the cytotrophoblastic invasion into the maternal vasculature [6,8], and involved in the placental and uterine artery remodeling [9,10]. In addition, increasing body of studies have suggested the use of MMP-2 or MMP-9 in the prediction or evaluation of PE [11–13], and even as drug targets [5]. However, the results were mixed and inconsistent with each other, therefore a more reliable predictor of PE is desperately needed.

In the present study, we aimed to investigate the reliability of MMP-2, MMP-9 and their relative ratio in the plasma to predict PE. Pregnant women with suspected PE were first recruited into the study, who were subsequently either cleared or diagnosed of HELLP syndrome or chronic hypertension with superimposed PE. Blood samples were collected from all participants, and plasma levels of MMP-2, MMP-9 and ratio of MMP-2/MMP-9 were analyzed with respect to the existence of PE conditions. We also investigated the predictive value of these factors using the receiver operating characteristic (ROC) curve analysis.

## Materials and methods

### Participant selection

Recruitment criteria in clinical signs and symptoms for suspected PE were adapted from previously established standards [14]: (i) new onset of elevated blood pressure (≥140 mmHg systolic and/or ≥90 mmHg diastolic); (ii) new onset of any protein in urine; (iii) aggravation of pre-existing hypertension; (iv) aggravation of pre-existing proteinuria; (v) low platelets, elevated liver transaminases, intrauterine growth restriction or abnormal uterine perfusion; (vi) epigastric pain, headache, excessive edema/severe swelling, visual disturbances or sudden weight gain (>1 kg/week).

During February 2012 to August 2015, a total of 318 pregnant women, who met at least one of the above inclusion criteria and were admitted to Shandong Provincial Qianfoshan Hospital, were initially recruited for the present study. All participants have given informed and written consent. The present study was approved by the ethics committee of Shandong Provincial Qianfoshan Hospital, and followed the ethical guidelines laid down in the 1975 Declaration of Helsinki.

Among the 318 pregnant women initially recruited, 25 participants were excluded due to withdrawal of consent or personal reasons during the study period. Among the rest of 293 study participants, 139 of them were confirmed to manifest HELLP syndrome or chronic hypertension with superimposed PE according to defined diagnostic standards [14]: (i) hypertension or gestational hypertension (≥140 mmHg systolic and/or ≥90 mmHg diastolic); (ii) proteinuria (≥0.3 g protein/24 h); (iii) HELLP syndrome (aspartate transaminase >70 IU/l, lactate dehydrogenase levels >600 IU/l, thrombocyte counts <100000/μl) [15]. The rest 154 pregnant women, who were not diagnosed of PE, were included in the study as normal pregnancy control group.

### Anthropometrical measurements

At the gestation age of 24 weeks, body weight was measured using a digital scale accurate to 0.1 kg. Body height was measured using a stadiometer accurate to 1 cm. The body mass index (BMI) was calculated as weight (kg)/square of height in metre-square.

### Biochemical analysis

At gestational age of 20 weeks, 10 ml of blood samples were collected from all study participants. Blood samples were collected in tubes with 0.1% EDTA and were centrifuged within 15 min of collection to separate the plasma. The plasma samples were stored at −80°C until further analysis. Plasma levels of MMP-2 and MMP-9 were measured using commercially available human MMP-2 and MMP-9 ELISA Kits (Sigma–Aldrich, St. Louis, MO, U.S.A.) following manufacturers’ protocols.

### Statistics

**Figure 1:**
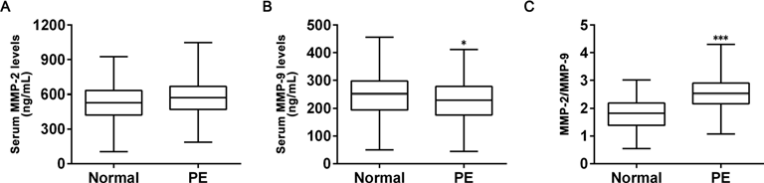
Change of plasma levels in PE group, including MMP-2 (A), MMP-9 (B), and MMP-2/MMP-9 ratio (C) of the participants in the two study groups. Values were presented as median and quartiles; *P<0.05, ***P<0.001 compared with normal pregnant.

All statistical analyses were performed using Prism 6 (GraphPad Software, Inc.). Values were presented as mean ± S.D. for Table 1, or median and quartiles for Figure 1. Two-tailed Student’s *t* test was used to analyze normally distributed data, and Mann–Whitney test was used to analyze non-normally distributed data. *P*<0.05 was considered as statistically significant.

**Table 1:** Baseline characteristics of the participants in the two study groups

Characteristics	Normal pregnant (*n*=154)	PE (*n*=139)
Age at pregnancy (year)	29.7 ± 4.8	31.2 ± 5.6
Gestational age at recruitment (week)	15.7 ± 3.2	16.3 ± 2.9
Gestational age at delivery (week)	39.6 ± 0.9	36.5 ± 2.1 *
BMI (kg/m^2^)	24.5 ± 3.7	25.1 ± 4.0
Systolic blood pressure (mmHg)	118.2 ± 9.6	153.5 ± 12.8 *
Diastolic blood pressure (mmHg)	75.9 ± 7.3	99.4 ± 8.2 *
Urinary protein (g/24 h)	0.25 ± 0.18	1.76 ± 0.25 *

Values were presented as mean ± S.D. BMI and blood pressure were measured at week 24; *P<0.05 compared with normal pregnant.

## Results

### Clinical characteristics

From February 2012 to August 2015, a total of 4426 pregnancy cases were admitted in Shandong Provincial Qianfoshan Hospital, among which 139 were diagnosed with HELLP syndrome or chronic hypertension with superimposed PE (PE group), making its incidence rate of 3.14%. Their clinical characteristics were analyzed and compared with 154 normal pregnant women (normal group) during the same period (see ‘Participant selection’ section), which was summarized in Table 1. While there were no differences in the age at pregnancy or gestation age at the time of recruitment between the two groups of study participants, gestational age at delivery was significantly earlier in PE group (36.5 ± 2.1 weeks) than in normal group (39.6 ± 0.9 weeks). Next, BMI of participants from both study groups were also found to be similar, at 24.5 ± 3.7 kg/m^2^ in normal group and 25.1 ± 4.0 kg/m^2^ in PE group (*P*>0.05). Hypertension was prevalent in participants from the PE group, with systolic blood pressure at 153.5 ± 12.8 mmHg and diastolic blood pressure at 99.4 ± 8.2 mmHg, both significantly higher than the normal group, showing systolic blood pressure at 118.2 ± 9.6 mmHg and diastolic blood pressure at 75.9 ± 7.3 mmHg respectively. Proteinuria was also common among participants of the PE group, with urinary protein of 1.76 ± 0.25 g/24 h, which was significantly higher than that of the normal group (0.25 ± 0.18 g/24 h).

### Plasma biochemical characteristics

In order to establish a predictive biomarker for accurate diagnosis of PE, we started with measuring the plasma concentrations of MMP-2 and MMP-9, both of which have been frequently implicated in PE-related studies [3,5,16]. Of note, none of the participants were taking anti-hypertensive drugs including calcium-channel blockers that might affect plasma levels of MMP-2 and MMP-9. First of all, plasma levels of MMP-2 were analyzed for participants from the normal and PE group (Figure 1A). We observed that MMP-2 level of PE participants was elevated than that of normal participants, but the difference was found to be statistically insignificant. On the other hand, plasma level of MMP-9 of PE participants was found to be slightly lower, but statistically significant (*P*<0.05), than that of participants from normal pregnant group (Figure 1B). Given our observations that MMP-2 and MMP-9 seemed to be regulated on opposite trends between normal pregnancies and those affected by PE, we were curious if a more prominent difference could be found, when we analyzed the ratio of MMP-2/MMP-9. In this context, we plotted MMP-2/MMP-9 ratio between the two study groups (Figure 1C), and indeed found that this ratio was markedly higher in PE group participants than those in normal pregnant group, and this difference was also statistically significant (*P*<0.001).

### Curves of ROC

**Figure 2:**
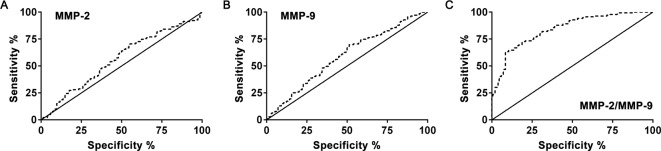
Predictive probabilities of plasma levels were analyzed by ROC curves Plasma levels include MMP-2 (A), MMP-9 (B) and the ratio of MMP-2/MMP-9 (C).

Furthermore, in order to investigate whether the MMP-2/MMP-9 ratio was indeed a more accurate biomarker of PE, the predictive values of MMP-2, MMP-9, and MMP-2/MMP-9 ratio were evaluated using ROC curve analysis. As shown in Figure 2A, we first analyzed the potential value of plasma level of MMP-2. We found that the area under the ROC curve for predicting PE was merely 0.563, with 95% confidence interval (CI): 0.497–0.629 (*P*>0.05). On the other hand, in the analysis for plasma level of MMP-9 (Figure 2B), the area under the ROC curve for predicting PE was found to be 0.587, with 95% CI: 0.522–0.652, which was statistically significant (*P*<0.05). Therefore, we further calculated the cut-off value of MMP-9 to distinguish PE-affected pregnancy, which was at 240 ng/ml, with 56.12% sensitivity (95% CI: 47.45–64.51%) and 55.84% specificity (95% CI: 47.63–63.83%). At last, the ratio of MMP-2/MMP-9 was subjected to the same ROC curve analysis (Figure 2C). The area under the ROC curve using MMP-2/MMP-9 ratio to predict PE was much higher than either MMP-2 or MMP-9 alone, at 0.841, with 95% CI: 0.797–0.886 (*P*<0.001). In addition, the cut-off value of using MMP-2/MMP-9 ratio to diagnose PE was 2.19, with 74.82% sensitivity (95% CI: 66.76–81.79%) and 74.68% specificity (95% CI: 67.05–81.33%).

## Discussion

In the present clinical study, we have recruited a total of 318 pregnant women with suspected PE-related symptoms, such as elevated blood pressure and/or proteinuria, among which 25 participants were excluded. The remaining participants initially enrolled into the study were later either clear or diagnosed of HELLP syndrome or chronic hypertension with superimposed PE, and were subsequently assigned as normal pregnant group (*n*=154) and PE group (*n*=139) respectively. We then compared the clinical characteristics and plasma biochemical parameters between the two study groups. We found there was essentially no difference with regard to age at pregnancy and BMI between the two groups of participants. However, gestational age at delivery of PE-affected participants was significantly earlier than normal pregnant group. In addition, PE-affected women also exhibited prominent hypertension and proteinuria, with systolic and diastolic blood pressures as well as urinary protein level much higher than the normal pregnant participants, which was consistent with their confirmed PE diagnosis.

Involvement of MMPs, particularly MMP-2 and MMP-9, in the pathogenesis of PE have been reported in various clinical studies. For instance, Myers et al. [17] reported that MMP-2 levels were increased in the plasma of women who later manifested PE, suggesting elevated net MMP-2 activity might contribute to PE pathophysiology. In another clinical study involving women undergoing genetic second-trimester amniocentesis, significantly elevated level of MMP-2 was detected in the amniotic fluid of women who eventually developed PE or superimposed PE, compared with normotensive women [11]. In the case of MMP-9, it was reported that its circulating levels were correlated negatively with diastolic blood pressure among patients with gestational hypertension [18]. Moreover, elevated levels of MMP-9 was observed in pregnancies that eventually developed PE [19]. In addition, Palei et al. [20] reported that both MMP-2 and MMP-9 levels could be affected by their polymorphisms, which were also associated with hypertensive disorders of pregnancy [21]. In fact, the same investigators found that higher net activity of MMP-9, but not MMP-2, was associated with gestational hypertension, however this trend in MMP activity was absent in PE [22]. In an animal study, MMP-9-deficient mice phenocopied PE symptoms of human patients such as intrauterine growth restriction and hypertension [13].

Given the above studies on the potential roles of MMP-2 and MMP-9 in predicting PE, we next measured the plasma levels of MMP-2 and MMP-9 for all participants, and compared their average levels between the two study groups. Data from our present study indicated that plasma level of MMP-2 in PE-affected participants was only increased by an insignificant margin compared with normal pregnant participants. In the case of MMP-9, we found a slight but significant decrease in women with PE. These data were largely in line with the above mentioned studies, although they were not strong enough to support the role for either of MMP-2 or MMP-9 alone as a predictive biomarker for suspected PE. Similar to our present study, plasma level of MMP-9 was found to be down-regulated in pregnancies that later developed HELLP syndrome [23]. A report by Montagnana et al. [12] suggested that MMP-2 levels were significantly higher in pre-eclamptic women, however, MMP-9 levels remained unchanged. Whereas Wang et al. [24] found that urine levels of both MMP-2 and MMP-9 were significantly higher before delivery in severe PE cases than normotensive groups. Moreover, Karampas et al. [25] found no difference in circulating MMP-9 concentrations between normal and PE pregnancies, which is different from results of our present study.

Taken together, results from the above-mentioned studies were inconsistent and sometimes even contradictory, although they collectively still suggested potential roles of MMP-2 and MMP-9 in PE, which calls for a more reliable predictive parameter involving perhaps both of them. In fact, studies involving simultaneous analyses of multiple factors have generated tangible biomarkers in predicting PE. Khosrowbeygi and Ahmadvand [26] have reported that the imbalance between the adipocytokines could be involved in the pathogenesis of PE, and the ratio of leptin/adiponectin was significantly increased in PE-affected women. In addition, the ratio of soluble Fms-like tyrosine kinase 1 (sFlt-1) to placental growth factor (PlGF) has been widely reported to reliably predict PE [27–31].

Inspired by these multi-factorial studies, we next calculated the individual ratio of MMP-2/MMP-9 for all study participants, and plotted them respectively to their PE status. As a result, we found this ratio was markedly and significantly higher in participants with PE than those without, which was not surprising due to their opposite trends of changes observed earlier. In order to further investigate the predictive value of MMP-2/MMP-9 ratio, we employed the ROC curve analysis to calculate the sensitivity and specificity in distinguishing PE from normal pregnancies. The result is promising in that, both the sensitivity and specificity of MMP-2/MMP-9 ratio are much higher and reliable than any one of them analyzed alone: the cut-off value of MMP-2/MMP-9 ratio is 2.19, with sensitivity and specificity of 74.82% and 74.68% respectively. This predicative value is at comparable level with that of sFlt-1/PlGF ratio, as Zeisler et al. [32] reported that sFlt-1/PlGF ≤38 had a considerably high negative predictive value of PE, with 80.0% sensitivity and 78.3% specificity. In line with the above data, analyzing both sFlt-1/PlGF and MMP-2/MMP-9 ratios among pregnant women could potentially provide even more comprehensive and reliable predictions for PE. Nevertheless, one important limitation of our present study lies in that, patients recruited were diagnosed of PE superimposed with HELLP syndrome or chronic hypertension, therefore factors that may contribute to either HELLP syndrome or chronic hypertension could interfere with interpretation of our data. Future studies, with larger cohort of patients so that enough patients with precise diagnosis can be enrolled, are needed for a more precise and comprehensive analysis, in order to validate the value of MMP-2/MMP-9 ratio in predicting PE.

To summarize, in the present clinical study, we aimed to investigate the reliability of using MMP-2 and MMP-9 as predictive biomarkers for suspected PE. Although any single MMP did not prove to be a promising candidate, the ratio of MMP-2/MMP-9 was found to be significantly elevated in PE-affected women. Further ROC curve analysis indeed supported the usefulness of MMP-2/MMP-9 ratio as an accurate biomarker in distinguishing PE from normal pregnancies with better sensitivity and specificity. Our study therefore demonstrates the predictive value of MMP-2/MMP-9 in women with suspected PE.

## Abbreviations

BMI: body mass index
CI: confidence interval
MMP: matrix metalloproteinase
PE: pre-eclampsia
PlGF: placental growth factor
ROC: receiver operating characteristic
sFlt-1: soluble Fms-like tyrosine kinase 1
